# Guided Depth Completion with Instance Segmentation Fusion in Autonomous Driving Applications

**DOI:** 10.3390/s22249578

**Published:** 2022-12-07

**Authors:** Mohammad Z. El-Yabroudi, Ikhlas Abdel-Qader, Bradley J. Bazuin, Osama Abudayyeh, Rakan C. Chabaan

**Affiliations:** 1 Electrical and Computer Engineering Department, Western Michigan University, Kalamazoo, MI 49008, USA; 2 Civil and Construction Engineering Department, Western Michigan University, Kalamazoo, MI 49008, USA; 3 Hyundai America Technical Center, Inc., Superior Charter Township, MI 48198, USA

**Keywords:** depth completion, instance segmentation, object detection, sensor fusion, LiDAR

## Abstract

Pixel-level depth information is crucial to many applications, such as autonomous driving, robotics navigation, 3D scene reconstruction, and augmented reality. However, depth information, which is usually acquired by sensors such as LiDAR, is sparse. Depth completion is a process that predicts missing pixels’ depth information from a set of sparse depth measurements. Most of the ongoing research applies deep neural networks on the entire sparse depth map and camera scene without utilizing any information about the available objects, which results in more complex and resource-demanding networks. In this work, we propose to use image instance segmentation to detect objects of interest with pixel-level locations, along with sparse depth data, to support depth completion. The framework utilizes a two-branch encoder–decoder deep neural network. It fuses information about scene available objects, such as objects’ type and pixel-level location, LiDAR, and RGB camera, to predict dense accurate depth maps. Experimental results on the KITTI dataset showed faster training and improved prediction accuracy. The proposed method reaches a convergence state faster and surpasses the baseline model in all evaluation metrics.

## 1. Introduction

Improvements in such domains as algorithms and sensors allowed self-driving cars (SDCs) to advance to commercial implementation and deployment in real-world testing. These SDCs utilize different types of sensors, such as color cameras, radars, LiDARs, ultrasonic sensors, and thermal cameras, for robust perception in real-life dynamic environments [1,2]. However, computing resources requirements increase as the number of sensors increases. Resource management and optimum utilization are critical to keeping SDCs operating in real-time. Currently, systems either use high-cost custom-processing units or compromise performance and utilize lightweight perception algorithms [3].

Among different perception tasks, depth perception is crucial for SDCs, robotics navigation, pose estimation, and trajectory prediction. LiDAR, RGB-D cameras, and stereo cameras can capture depth information. LiDAR is considered the most accurate depth sensor currently available on the market, which can operate in indoor and outdoor scenarios and provide precise depth information for near and far objects [4,5,6]. LiDAR data can be represented in three formats: voxels, meshes, and point clouds [7,8,9]. A voxel-based representation can apply a traditional convolutional neural network (CNN) to 3D data. However, for high resolution, the storage and computing resources required by the voxel method significantly increase, making it unsuitable for high-resolution point cloud reconstruction. On the other hand, the mesh is a surface representation derived from a point cloud by sampling a set of vertices and defining faces between these points. A point cloud format is more straightforward, more efficient for 3D shapes, and easier to manipulate when geometric transformations are needed [8].

**Figure 1:**
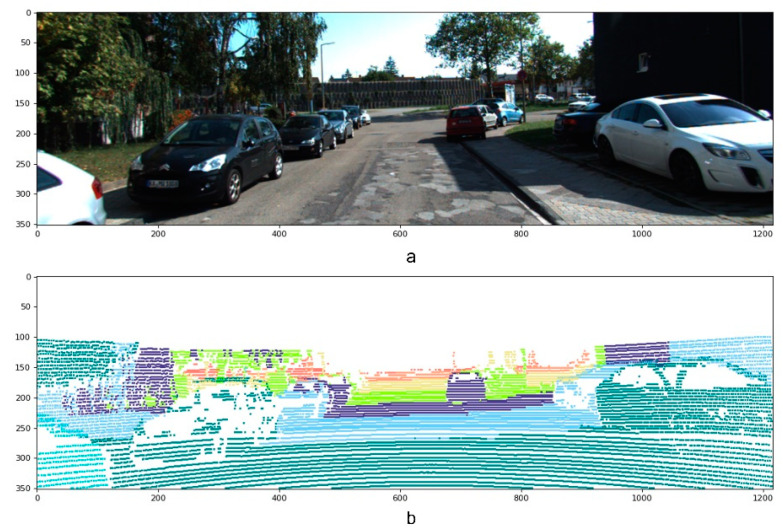
Sparse point cloud example: (**a**) reference RGB image; (**b**) sparse depth map color-encoded and enlarged for better visualization.

Due to the physical assembly and technology that LiDAR uses, the generated depth information is sparse. When projected into RGB images, it only provides 5% valid depth information, which means a severe loss in geometric details and, consequently, restricting dependent processes [10,11,12]. Figure 1 shows an example of the sparse LiDAR measurements alongside the reference RGB image. It is worth mentioning that the sparse depth map has been colored and enlarged for better visualization.

The performance of the applications that depend on LiDAR information suffers significantly from the sparsity of data, and perception tasks, such as 3D object detection and obstacle avoidance, require dense depth information [5,6,13].

The depth completion process tackles the problem of estimating dense depth information from a sparse, incomplete set of depth measurements, usually with the help of RGB images. Depth completion methods fall into two categories: depth-only and image-guided methods. Dense depth maps are generated directly from the sparse, raw LiDAR data in depth-only methods. In image-guided methods, textural information from RGB images is utilized for more accurate depth estimation. Most of the ongoing research applies depth completion techniques on the entire field of view and designs complex learning models to improve depth estimation in challenging scenarios, such as when objects are small or far away, or to enhance depth measurements at objects’ boundaries [14,15]. However, to our knowledge, none of the existing work has integrated prior knowledge about scene objects or utilized pre-processes that generate additional features that can guide the depth completion process with simpler deep networks. We believe that fusing more clues to the input of deep neural networks can result in better performance without creating very complex and resource-intensive networks.

**Figure 2:**
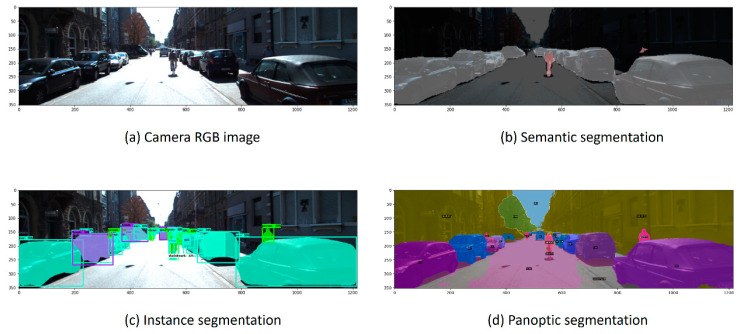
Image segmentation methods [18].

In computer vision, image segmentation falls into three main methods: semantic, instance, and panoptic. Semantic segmentation is the process of assigning class labels for every pixel (e.g., people vs. background). Instance segmentation provides instance information for each pixel, which means it first detects all the objects of interest in an image and then assigns unique labels and IDs for each pixel. Panoptic segmentation combines both instance and semantic segmentation and generates labels for each pixel in a digital image while distinguishing between different instances from the same class [16,17]. Figure 2 shows three examples of the three different segmentation methods. Instance segmentation is unique because it can focus on a pre-selected set of objects within a scene and generate accurate pixel-level object masks and types. Moreover, most of the current SDC frameworks are already utilizing it for other purposes, including object localization. Thus, instance segmentation information will be free-of-charge features that can help the depth completion pipeline.

To address these challenges, we designed a framework that integrates vision data with LiDARs using fusion approaches to enhance depth completion while using a relatively noncomplex deep neural network. The core contributions of this work are as follows: (1) design of a data structure and encoding scheme to reduce instance segmentation disk storage requirements for faster training; (2) fusion of instance segmentation features into the learning pipeline; (3) fusion of pixel-level object masks for better depth estimation at objects’ boundaries; and (4) fusion of object type information for better depth estimation within objects.

## 2. Related Work

Recent depth completion methods can be classified into two main categories: guided and non-guided approaches. The guided techniques utilize auxiliary information, such as RGB information, to guide the depth completion process, while the non-guided techniques rely only on sparse depth measurements. It is worth mentioning that guided approaches usually produce better results and, thus, are commonly used [14]. Researchers have developed various techniques, primarily convolutional neural network (CNN) solutions. We briefly review these techniques in this section.

### 2.1. Non Guided Techniques

In non-guided depth completion techniques, only LiDAR data are utilized. Premebida et al. [19] used local interpolation to estimate each pixel location within the sparse depth image. The authors analyzed different non-deep learning reconstruction techniques that rely on depth information, such as inverse distance weighting, Shepard’s method, ordinary Kriging, Delaunay triangulation, and bilateral filtering. In addition, they introduced a modified bilateral filter that handles the depth dispersion within the interpolation area. Uhrig et al. [11] proposed a complete deep neural framework with sparse convolution layers that take the location of missing data to tackle the depth completion problem and only rely on the sparse LiDAR information. Eldesokey et al. [20] proposed CNNs that focus on the uncertainty of depth data in both the input and the output; the work uses an input confidence estimator to identify distributed measurements in the input. Moreover, a normalized convolutional neural network is utilized to produce an uncertainty measure for the final output. Chodosh et al. [21] used compressed sensing techniques and alternating direction neural networks (ADNN) to tackle the depth map completion problem. The adoption of ADNNs enabled implementation of a deep recurrent encoder–decoder framework.

### 2.2. Guided Techniques

**Figure 3:**
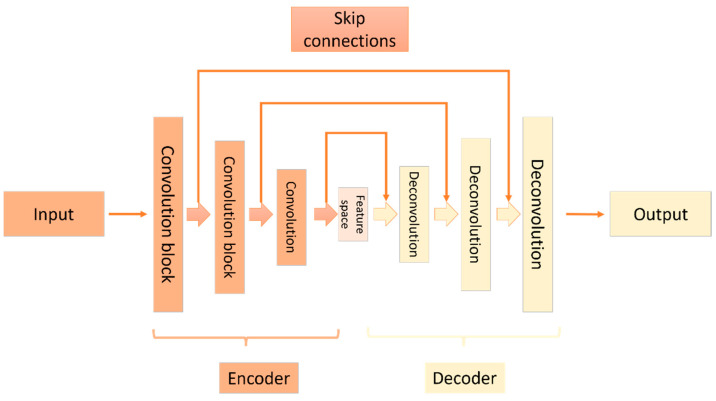
Encoder–decoder deep neural network architecture with skip connections.

Fusing information from multiple related modalities has led to remarkable performance enhancement in a wide spectrum of applications [6]. Many fusion strategies have been proposed. Various works suggest combining data from multiple sensors, such as LiDAR and cameras, for depth completion problems. Ma and Karaman [22] considered depth completion a regression problem. They employed a single convolutional neural network that utilizes RGB images and a sparse depth measurement as input that generates a dense depth map. The proposed network is an encoder–decoder architecture wherein the encoder is a residual neural network (ResNet) CNN followed by a convolutional layer. On the decoder side, up-sampling layers are followed by bilinear up-sampling. The work evaluated different loss functions and reported that the least absolute deviation loss produced better performance. Ma et al. [23] proposed a deep regression network with an encoder–decoder style. Data from LiDAR and the camera are fused within the network. A skip connection is used to pass features from the encoding layers to the corresponding decoding layers. Figure 3 depicts a general structure of an encoder–decoder deep neural network with skip connections. These connections are beneficial for passing features from encoder layers directly to the corresponding decoder layers; this process provides the decoder layers with additional information that might be degraded during the encoding process. In addition, the authors also proposed a self-supervised training framework that relies only on a sequence of RGB images and sparse depth images. That is, no ground truth is needed. The existence of nearby data is used to provide supervision signals. Hu et al. [24] provided a two-branch encoder–decoder framework with geometric encoding and multiple levels and modalities fusion, where the authors fuse RGB and LiDAR data in one branch and fuse the generated semi-depth with the depth in another branch. Moreover, features are fused at different levels and between the two branches.

Qiu et al. [25] introduced DeepLiDAR framework, which consists of two separate pipelines. The first pipeline generates surface normal from a sparse set of measurements. The second pipeline is used to obtain a semi-depth map from RGB images, and, finally, surface normal and semi-depth maps are fused and fed into another network trained to produce the final depth map. Neven & Leuven [26] proposed FusionNet, a two-branch framework, one branch for local features and the second for global features. FusionNet generates confidence maps to fuse information from different branches properly. Xiong et al. [27] suggested a more accurate sampling strategy and proposed a deep neural network with a graph convolution module to overcome the limitations of the traditional square kernel.

Another promising deep neural network architecture is generative adversarial network (GAN). This architecture consists of two separate models, a generator and a discriminator. The generator network learns from the input distribution and tries to generate perfect output. On the other hand, the discriminator uses ground truth to assess the generator’s performance. Recently, some work utilized the GAN networks to complete the sparse depth information. Zhang et al. [28] proposed a multitask generative adversarial network that works for both semantic segmentation and depth completion; they used the semantic segmentation output as input to improve the depth prediction accuracy. This work is guided as it uses the RGB images alongside the sparse LIDAR depth information. The authors introduced multi-scale pooling blocks within the network to extract features from different levels. The architecture has two main branches, one for semantic segmentation and the other for depth completion, which yields two generators and two discriminators. Nguyen and Yoo [29] proposed GAN architecture with an encoder–decoder generator network similar to [24]. However, for the generator inputs, the authors examined the impact of semantic segmentation and further applied anisotropic diffusion for post-processing and smoothing.

### 2.3. Image Segmentation

Algorithms that tackle image segmentation tasks are numerous and can be grouped into two main categories: early non-deep learning methods, such as thresholding, region-growing graph cuts, and active contours, and deep learning methods, which, in recent years, have produced impressive performance enhancements and paradigm shifts in the image segmentation field [16]. Since 2014, many deep-neural-network-based image segmentation algorithms have been proposed; the most popular ones fall into two categories: two-stage and one-stage. In the two-stage category, algorithms perform two subtasks: detection and segmentation. Depending on the order of these subtasks, two-stage instance segmentation can further be divided into two methods: top-down and bottom-up. The former is a detection-based instance segmentation method wherein top-level bounding boxes are first generated. Foreground segmentation is conducted, while the latter is a segmentation-based method that starts with pixel-level segmentation and then uses clustering to group similar pixels. Examples of instance segmentation methods are Mask R-CNN [30], InstanceFCN, FCIS, TensorMask and YOLACT, SOLO, SOLOv2, and CenterMask [31].

## 3. Materials and Methods

In this research, we selected the Mask-RCNN instance segmentation architecture to find the instance segmentation of each RGB image within the KITTI dataset. Mask R-CNN is a two-stage architecture that allows for better control over each part individually. Moreover, it is a stable and supported architecture. Although there are available pre-trained Mask R-CNN models already trained, we noticed that, on the KITTI dataset, these pre-trained models do not work well. Therefore, we used transfer learning to adapt a pre-trained COCO model to work better on the KITTI depth dataset for better detection. We used the KITTI instance segmentation dataset and performed transfer learning.

To reduce the training time, we prepared the instance segmentation dataset for the entire depth dataset offline and then used it in the training process. Although the trained instance segmentation model can detect many objects in the scene, we focused on the three main objects: cars, pedestrians, and cyclists. The output of the Mask-RCNN is presented in the following format:

- List of all detected objects numerically encoded (e.g., {0, 0, 0, 1, 1, 2}).
- List of detection confidence (e.g., {0.99, 0.85, 0.51, 0.90, 0.70}).
- For each object, a 2D mask array of the same size as the input RGB image. For example, Mask R-CNN will return *N* × (*W* × *H*) arrays, where *N* is the number of detected objects.
- Bounding box information (ROI) for each detected object.

The previous list is huge, especially the third item, because it dramatically increases the storage requirement (e.g., if a single mask array requires 1 MB of storage, then an image with five objects requires 5 MB). We designed a data structure and encoding scheme to solve this problem that dramatically reduced storage requirements. We combine all object masks into one single global mask. To distinguish between different objects within the global mask, we utilize the object IDs; thus, our final global mask is no longer binary; it contains other numerical numbers representing different objects.

**Figure 4:**
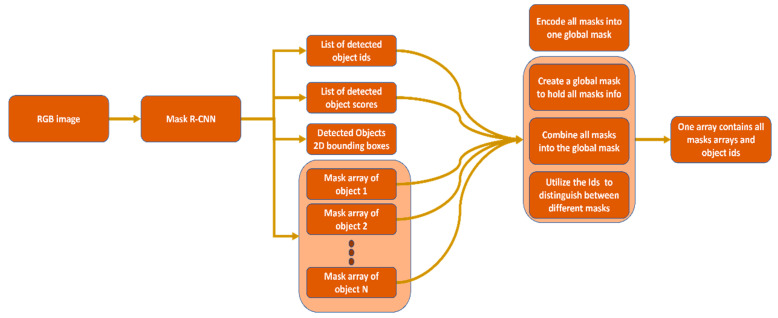
Mask R-CNN output and the encoding process integration; the encoding process will utilize the object IDs and combine all masks into a single global mask.

Algorithm 1 describes the primary operation of the proposed encoding scheme. In summary, the algorithm creates an array that internally will hold three sub-arrays: (1) an array of the objects’ IDs, (2) an array of detection scores, and (3) an array that contains the aggregated masks for all detected objects. To prepare the third array, the algorithm loops over Mask R-CNN objects’ masks and combines them into a single array. Figure 4 depicts the integration between the Mask R-CNN output and the encoding algorithm. This algorithm will generate a small footprint of the needed instance segmentation information. In fact, the physical memory requirement is dramatically decreased. Table 1 shows a physical memory storage requirement comparison between the original Mask-RCNN output and the encoded version.

**Figure 5:**
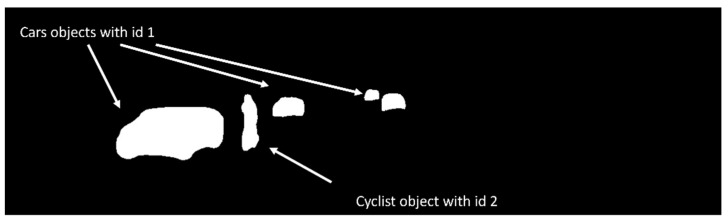
Instance segmentation objects’ masks and types encoded into a single 2D 1-channel array.

In training, instance segmentation information is prepared in the shape of a 2D image, where pixels corresponding to potential objects are assigned the object class ID value. In contrast, other pixels will have a zero value. Figure 5 depicts an example of the encoded instance segmentation feature masks and object types into a single 2D 1-channel array for a randomly selected fram from the KITTI dataset. In the figure, cars’ pixels were assigned an ID value of 1, cyclist pixels were assigned an ID value of 2, and all nonrelevant objects were assigned an ID value of 0.
**Algorithm 1**: algorithm to encode instance segmentation masks and objects type **Function**Encode_instance(i)**Input**i: array—RGB image with size (w,h,c)**Output**r: array with size (w,h,1)1://calling Mask R-CNN instance segmentation model2:R = instance_segmentation_Detect(i)

3://Create empty arrays to hold the output4:Fram_instance_seg_info = []5:masks_idx = []

6://append all class ids to the output array 7:fram_instance_seg_info.append(R[‘class_ids’])8://append all detection scores to the output array9:fram_instance_seg_info.append(R[‘scores’])

10://loop over all detected objects11:for j in range(len(R[‘class_ids’])):12:obtain indexes of the pixels belonging to objects13:mask_idx = np.where(R[‘masks’] [j] == True)

14://append indexes to the array 15:masks_idx.append(mask_idx)



16://append the full masks indexes array to the output array 17:fram_instance_seg_info.append(masks_idx)

18:return fram_instance_seg_info



### 3.1. Problem Formulation

Depth completion deals with the problem of estimating a dense set of depth measurements from a sparse input. Let us assume the dense output is *D* and the sparse input *S*; then, *D* can be estimated using a network *N* with parameters θ as formulated in Equation (1):

(1) D=N(S, θ)

Equation (1) applies to non-guided depth completion problems where only a sparse LiDAR point cloud is used. However, accurate dense depth maps can be obtained by combining multi-sensor information. A commonly used method is fusing data from RGB cameras and LiDAR in early or late fusion mechanisms. This technique is formulated in Equation (2) for RGB image I:

(2) D=N(S, I, θ)

In this research, we updated this formula and added other exciting information to improve the guided depth completion performance. Instance segmentation pipelines provided useful information, such as object types, objects locations in a bounding box format, and pixel-level mask for detected objects. This information is very beneficial in guiding the DNN. Thus, we revisited Equation (2) and included instance segmentation information—object type and object mask—as in Equation (3):

(3) D=N(S, I, M, T, θ)

where M and T represent the object’s pixel-level mask and object types, respectively; thus, network *N* will combine interesting features from the sparse depth map, the RGB image, the pixel-level object locations, and the object types.

Parameter θ is optimized during training by minimizing the loss function given a ground truth sample gt as in Equation (4):

(4) $$\hat{\theta }=\mathrm{argmin}\;ℒ\;\left(D,\;\mathrm{gt}\right)$$

where ℒ, $\hat{\theta },\;\mathrm{gt}$ represents the loss function, network parameters, and the ground truth sample, respectively. In Equation (4), each pixel depth value in the generated depth map D will be compared to the corresponding depth value in the ground truth depth map; the smaller the difference, the better.

We used the $l_{2}$ loss (squared error loss), which squares the difference between the prediction value and the ground truth as in Equation (5).

(5) $$l_{2}=\overset{n}{\underset{i}{\sum }}{\left(D-gt\right)}^{2}$$

### 3.2. Depth Completion and Fusion Network Architecture

The most recent state-of-the-art work adopted deep neural network methods, specifically convolutional neural networks with skip connections and encoder–decoder architecture similar to the architecture depicted in Figure 3. In this work, we assume a similar trend and utilize a multi-branch encoder–decoder network that takes the sparse depth map, the RGB image, and the instance segmentation data as input and produces a denser depth map as an output. We also adopt multi-level features fusion, early and late. Skip connection is also utilized to preserve the small details that could diminish while the encoder encodes the input data into smaller feature space.

**Figure 6:**
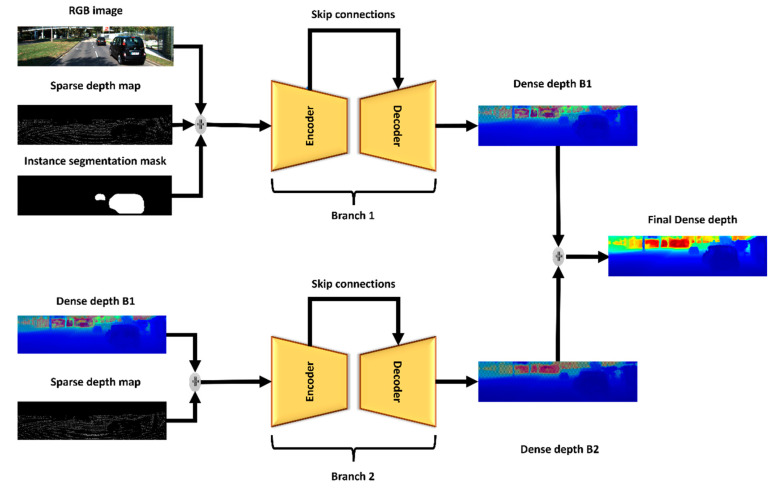
The network architecture of the proposed method with three distinct inputs: RGB image, the sparse depth map, and instance segmentation features, and two encoder–decoder branches with skip connections. Depth maps are color encoded for better visualization. Cold colors represent near objects, while warm colors represent distant objects.

Figure 6 depicts the used network architecture; the network consists of two similar branches; the input for the first branch is a concatenation between the sparse depth map, the RGB image, and the instance segmentation. The input for the second branch is a concatenation between the sparse depth map and the semi-dense depth map generated from the first branch. The encoder consists of a convolutional layer and ten residual blocks [32]. The decoder comprises six deconvolutional layers; more information about the network configuration is provided in Table 2. The decoder will try to reconstruct the depth values and then compares the results with the ground truth to update learning parameters. Similarly, the second branch obtains the output from the first branch, fuses it with the sparse depth values, and forwards the results to branch 2, which has a similar configuration to branch 1.

It is noteworthy to mention that the network employs different levels of data fusion. First, instance segmentation masks and object types encoded using our encoding technique are concatenated with the sparse depth map and the RGB images. Second, the generated features from each residual layer are bypassed to the relevant deconvolution layer at the decoder segment throughout the skip connection approach. Third, the output of the first branch is concatenated with the sparse depth map and fed into the second branch. Finally, the dense depth maps generated from branches one and two are fused adaptively. We follow the same fusion strategy in [26] as in Equation (6).

(6) $${\hat{D}}_{Fused}\left(u,v\right)=\frac{e^{C_{B1}\left(u,v\right)}.\;{\hat{D}}_{B1}\left(u,\;v\right)+e^{C_{B2}\left(u,v\right)}.\;{\hat{D}}_{B2}\left(u,\;v\right)}{e^{C_{B1}\left(u,v\right)}+e^{C_{B2}\left(u,v\right)}}$$

where $C_{B1}\left(u,v\right)$, $C_{B2}\left(u,v\right)$, ${\hat{D}}_{B1}\left(u,\;v\right)$, ${\hat{D}}_{B2}\left(u,\;v\right)$ represent the confidence map from the first branch, the confidence map from the second branch, the estimated depth from the first branch, and the estimated depth from the second branch, respectively, and (*u*,*v*) represents the pixel location.

## 4. Experimental Work

This section describes the implementation details of the proposed instance-segmentation-based depth completion framework. Then, we present a quantitative and qualitative evaluation on the KITTI depth completion benchmark dataset [18].

### 4.1. Dataset and Evaluation Metrics

In this research, we used the KITTI depth completion dataset [18], which consists of 85,898 training data frames from RGB cameras and LiDAR; the dataset also has 1K validation data frames. We observed that the RGB images are extracted from two cameras positioned to capture the car’s front view. To reduce the training time, we used only data from one camera, which reduced the number of frames to 42,949, and, further, we applied ¼ random sampling, resulting in a final training dataset size of 10,737 frames. Each training sample consists of four main entities: (1) RGB frame with a resolution of 1216 × 352, (2) sparse LiDAR depth map, (3) ground truth depth map, and (4) instance segmentation information encoded using our encoding algorithm. It is worth noting that the sparse depth maps have about 5% valid depth information, and the ground truth depth maps have about 16% valid depth information [33].

For evaluation, we followed the most commonly used metrics: root mean squared error (RMSE), mean absolute error (MAE), root mean squared error of the inverse depth (iRMSE), and mean absolute error of the inverse depth (iMAE), formulated in Equations (7), (8) (9) and (10), respectively [14].

(7) $$\mathrm{RMSE}=\sqrt{\frac{1}{N}\;\overset{N}{\underset{i}{\sum }}{\left({\hat{d}}_{i}-d_{i}\right)}^{2}}$$

(8) $$\mathrm{MAE}=\frac{1}{N}\;\overset{N}{\underset{i}{\sum }}\left|\left({\hat{d}}_{i}-d_{i}\right)\right|$$

(9) $$\mathrm{iRMSE}=\sqrt{\frac{1}{N}\overset{N}{\underset{1}{\sum }}{\left|\frac{1}{{\hat{d}}_{i}}-\frac{1}{d_{i}}\right|}^{2}}$$

(10) $$\mathrm{iMAE}=\frac{1}{N}\;\overset{N}{\underset{1}{\sum }}\left|\frac{1}{{\hat{d}}_{i}}-\frac{1}{d_{i}}\right|$$

### 4.2. Experimentation Environment

As described in Figure 5, the proposed method is implemented using the Pytorch framework. The training and evaluation processes have been conducted using a Google Colab environment with Pro membership, which provides Tesla P100 or Tesla T4 GPU with 25.4 GB graphic RAM and 32 GB of machine RAM and 167 GB SSD storage. To assist the training, we use Adam optimizer with parameters 0.9 and 0.999 for $\beta_{1}\;$and $\beta_{2}$, respectively, and with a weight decay of $10^{-6}$. The model has been trained for 15 epochs with a batch size of 4 and a learning rate of 0.001. Our baseline model is the ENet [24] model. Since we are using a subset of the KITTI dataset, we first retrained the ENet on the prepared subset and used it as our baseline.

### 4.3. Experimental Results

#### 4.3.1. Instance Segmentation Transfer Learning

**Figure 7:**
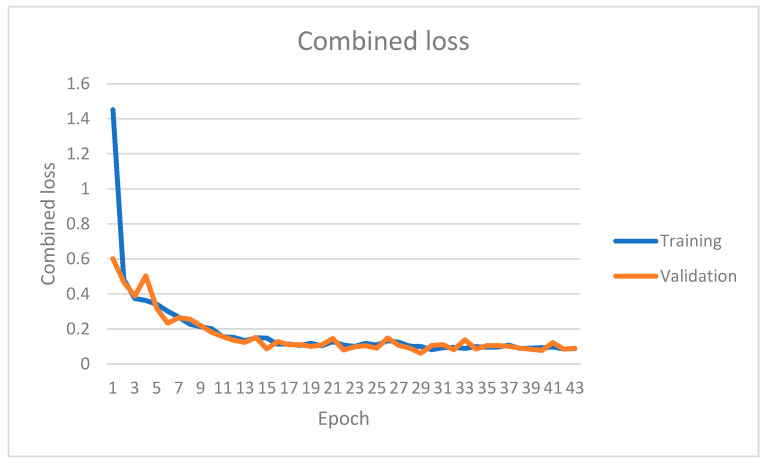
Instance segmentation combined loss on both the training and validation datasets over different epochs.

**Figure 8:**
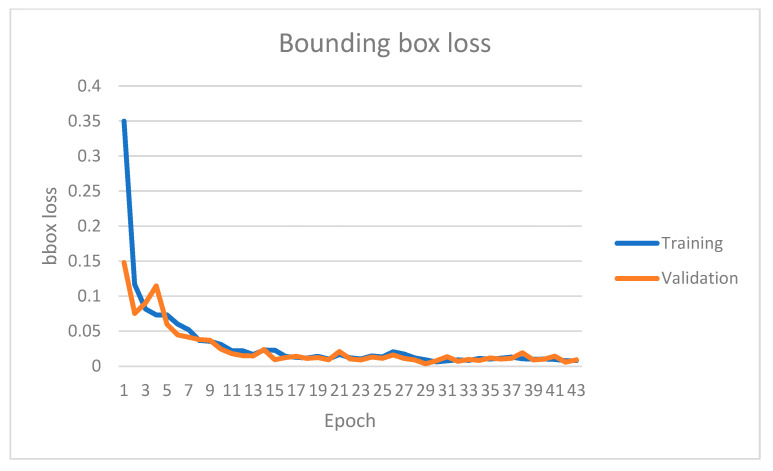
Instance segmentation bounding box loss on both training and validation datasets over different epochs.

**Figure 9:**
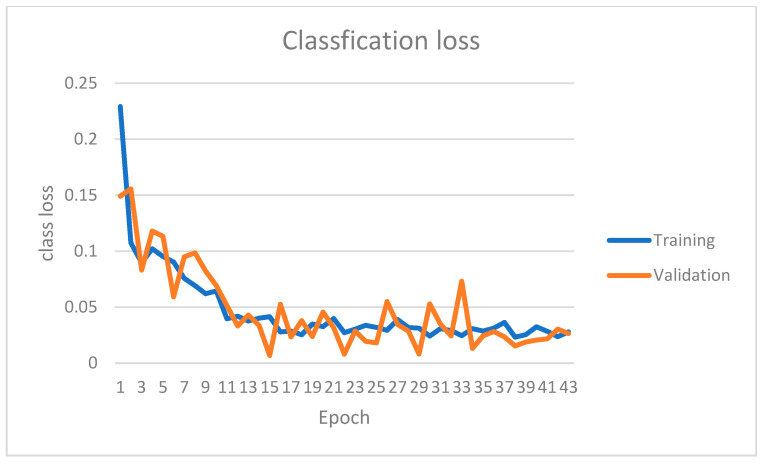
Instance segmentation classification loss on both training and validation datasets over different epochs.

**Figure 10:**
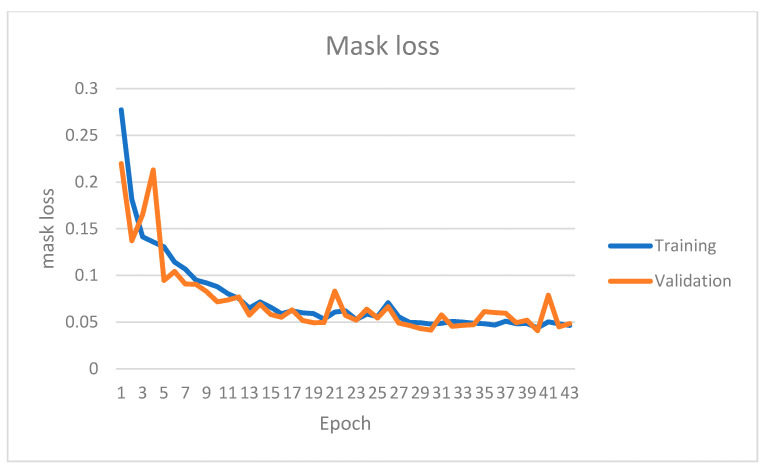
Instance segmentation mask loss on both training and validation datasets over different epochs.

The instance segmentation network has been trained on the KITTI instance segmentation dataset for 42 epochs. Figure 7, Figure 8, Figure 9 and Figure 10 show four primary loss performances over the epoch’s interval: the combined loss, bounding box loss, class loss, and mask loss, respectively. Loss values were recorded for both the training subset and validation subset. All losses decrease significantly within the first 30 epochs. For example, the training mask loss was around 0.06 at epoch 30 and stayed around the same value for the remaining training process. The validation loss is generally small and very close to the training loss, which means that the model is not overfitted and can generalize well for new unseen data.

**Figure 11:**
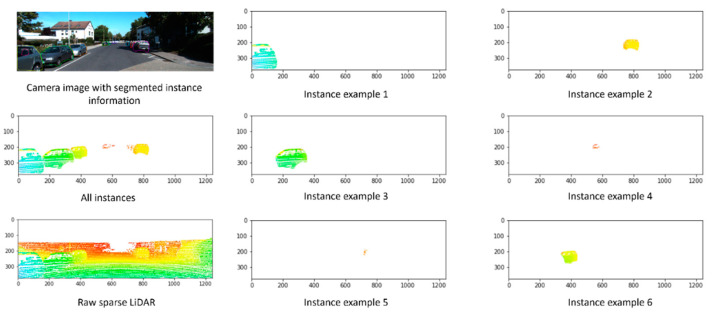
Performance of the trained instance segmentation model on a randomly selected frame from the KITTI dataset. Objects’ distance is color encoded; near objects have cold color while distant objects have warm colors.

**Figure 12:**
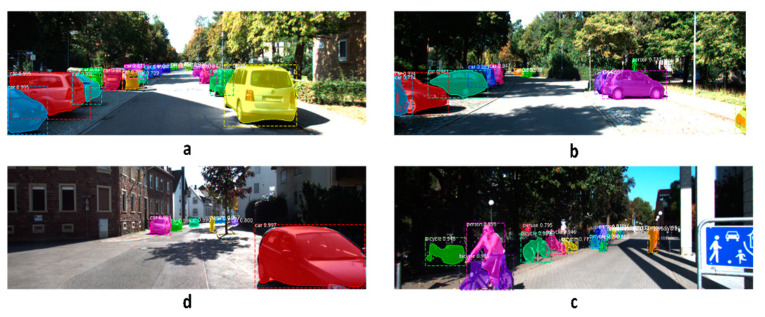
Qualitative results of the trained instance segmentation model on randomly selected frames from the KITTI dataset. (**a**,**b**) show the performance in a crowded vehicle scene, (**c**) shows the performance when objects are very far, and (**d**) shows the performance in a cyclists’ crowded scene.

Figure 11 shows the performance of the instance segmentation model on a randomly selected frame from the KITTI dataset. The top left image shows the frame with the instance mask placed on each object. The figure also shows the LiDAR sparse point cloud and instances examples. Depth completion neural networks can work on each instance individually or the entire scene with preliminary information about instance masks and types. Figure 12 shows four different qualitative results from the trained instance segmentation model for a variety of road objects, such as cars, pedestrians, and cyclists. The trained instance segmentation model was able to accurately detect and localize all relevant road objects, such as cars, pedestrians, and cyclists. Far, small, and occluded objects were also accurately detected.

#### 4.3.2. Depth Completion Network

**Figure 13:**
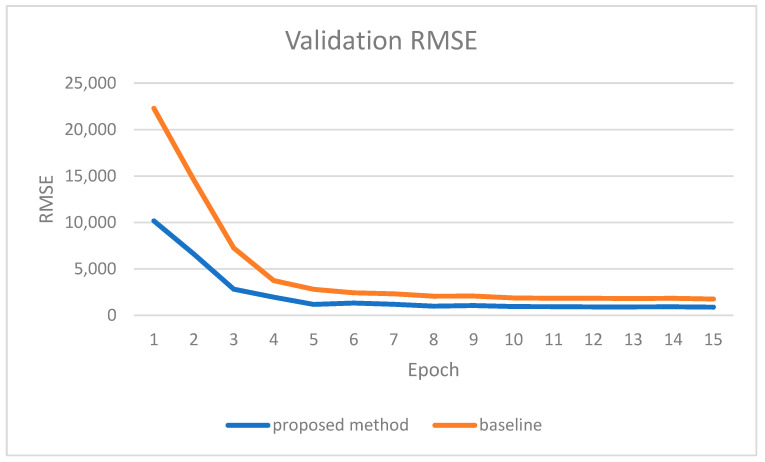
Validation RMSE for each epoch for both the proposed method and the baseline.

We evaluated the proposed method using the standard evaluation metrics formulated in Equations (7)–(10). Interestingly, the proposed method surpasses the baseline model in all metrics. Moreover, we noticed a significant margin of error between the proposed method and the baseline model, especially in early epochs. In Figure 13, we report the validation RMSE, which is usually used as the primary metric to evaluate depth completion performance. An interesting observation in the figure is the considerable margin between the proposed method and the baseline model at the first epochs. This confirms instance segmentation’s positive impact on guiding the depth completion network.

**Figure 14:**
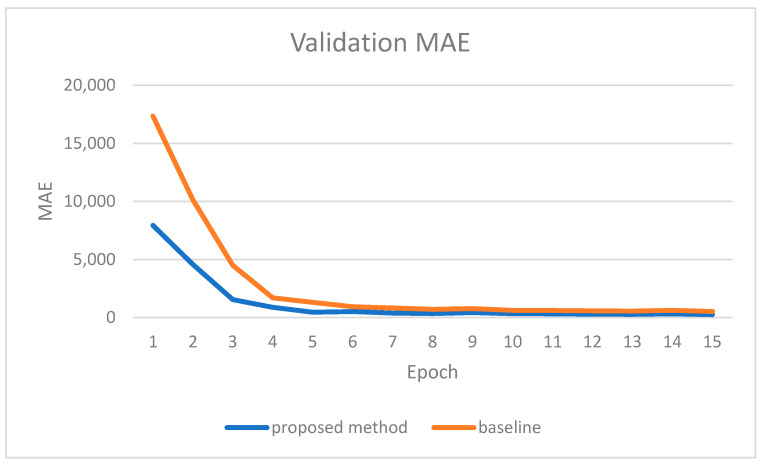
Validation MAE for each epoch for both the proposed method and the baseline.

We noticed the same trend in other parameters. Figure 14 shows the validation MAE for each epoch. As with the RMSE, MAE also started with a smaller value for the proposed method than the baseline model. Table 3 summarizes the evaluation results on the KITTI dataset for both the baseline model and the proposed method at the last epoch of training. The proposed method surpasses the baseline model in all evaluation metrics, and this performance improvement was expected. The proposed method employed handy features from the instance segmentation output, which are the object types and the pixel-level objects’ locations. Both features are significant in boosting deep neural network learning capabilities. The deep neural network can utilize the object types to more accurately estimate depth values on the objects’ surfaces. Similarly, the deep neural network utilizes the accurate objects’ locations to precisely estimate the depth at objects’ boundaries, especially when objects are very close to each other.

**Figure 15:**
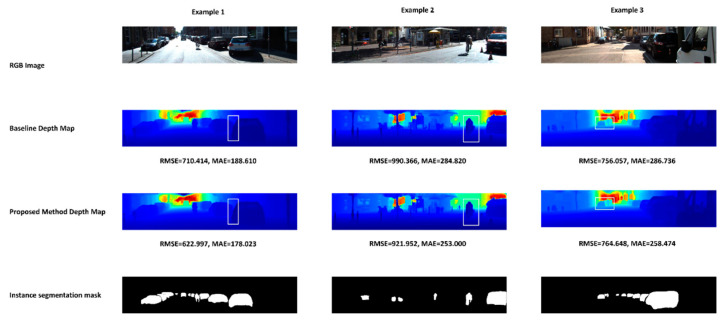
Qualitative results for randomly selected samples. The white rectangle inside depth maps images indicates the interesting areas.

Figure 15 shows qualitative results for three randomly selected examples. For each example, we are providing the RGB image as a reference, as well as the proposed method’s depth map, the baseline model’s depth map, and the instance segmentation mask. We are also highlighting interesting areas that are easy to focus on to see the strength of the proposed method compared to the baseline model. Finally, we also provide the RMSE and MAE for each example. In example one, we are highlighting the area between two adjacent cars; the provided instance segmentation mask clearly distinguishes between the two individual cars; in the proposed method, the boundaries of both vehicles are sharp, and even the area between the cars is more visible. However, in the baseline model, the car and the area between the two cars merged and assigned similar depth values. In the second example, we highlight the cyclist; the boundaries’ depth values are more accurately estimated in the proposed method. In the baseline model, the right side of the cyclist’s body is less sharp and mixed with the background. In the third example, we highlight two pedestrians walking together; the proposed method provided accurate depth for each person individually and even for the area between them. On the other hand, the baseline model assigned inaccurate depth values between the two pedestrians.

## 5. Conclusions

In this paper, a framework has been proposed and implemented that uses image instance segmentation, sparse LiDAR data, and RGB images to generate dense depth maps with object-level consideration. An encoding algorithm was introduced for proper working with instance segmentation features, wherein both objects’ masks and IDs are fused into a single 2D array with a single channel. The trained network maintains performance superior to the baseline model in all evaluation metrics. Additionally, in earlier epochs, the experimental results show the ability of the proposed method to start with smaller error than the baseline model, which reveals two important observations. First, instance segmentation features can play a significant role in guiding depth completion. Second, the ability of the network to reach a convergence state faster with fewer training epochs is achievable. The proposed framework can be generalized and utilized to fuse other sensor data, such as RADARs, stereo vision, and thermal cameras, and thus be able to fuse LiDAR sparse data with additional features extracted using algorithms best fitted to these sensors’ data for better perception and scene understanding in future work.

## Figures and Tables

**Table 1:** Comparison between the raw Mask R-CNN physical memory needs and the encoded Mask R-CNN physical memory needs for 300 frames.

Metric	Mask R-CNN	Encoded
Memory footprint	13.00 GB	1.04 GB

**Table 2:** Depth deep neural network encoder and decoder configuration.

	Layer	Type	Output Shape	Kernel Size	Stride	Padding	With BN	Activation
	0	Conv	(32,352,1216)	5	1	2	True	ReLU
**Encoder**	1	Residual	(64,176,608)	3	2	1	True	ReLU
	2	Residual	(64,176,608)	3	1	1	True	ReLU
	3	Residual	(128,88,304)	3	2	1	True	ReLU
	4	Residual	(128,88,304)	3	1	1	True	ReLU
	5	Residual	(256,44,152)	3	2	1	True	ReLU
	6	Residual	(256,44,152)	3	1	1	True	ReLU
	7	Residual	(512,22,76)	3	2	1	True	ReLU
	8	Residual	(512,22,76)	3	1	1	True	ReLU
	9	Residual	(1024,11,38)	3	2	1	True	ReLU
	10	Residual	(1024,11,38)	3	1	1	True	ReLU
**Decoder**	11	DeConv	(512,22,76)	5	2	2	True	ReLU
	12	DeConv	(256,44,152)	5	2	2	True	ReLU
	13	DeConv	(128,88,304)	5	2	2	True	ReLU
	14	DeConv	(64,176,608)	5	2	2	True	ReLU
	15	DeConv	(32,352,1216)	5	2	2	True	ReLU
	16	DeConv	(2,352,1216)	5	2	2	True	ReLU

**Table 3:** Evaluation metrics for the last trained epoch using the validation dataset where ↓ indicates that the lower the value is the better).

Metric	Baseline	Proposed Method
RMSE ↓ (mm)	882.636	879.525
iRMSE ↓(1/KM)	3.178	3.1585
MAE ↓(mm)	266.933	261.991
iMAE ↓(1/KM)	1.279	1.262

## Data Availability

Publicly available datasets were analyzed in this study. These data can be found here: https://www.cvlibs.net/datasets/kitti/ (accessed on 16 October 2022).
